# Fibromyalgia: Anti-Inflammatory and Stress Responses after Acute Moderate Exercise

**DOI:** 10.1371/journal.pone.0074524

**Published:** 2013-09-04

**Authors:** Maria Elena Bote, Juan Jose Garcia, Maria Dolores Hinchado, Eduardo Ortega

**Affiliations:** Department of Physiology, University of Extremadura, Badajoz, Spain; University of Sao Paulo, Brazil

## Abstract

Fibromyalgia (FM) is characterized in part by an elevated inflammatory status, and “modified exercise” is currently proposed as being a good therapeutic help for these patients. However, the mechanisms involved in the exercise-induced benefits are still poorly understood. The objective was to evaluate the effect of a single bout of moderate cycling (45 min at 55% VO_2_ max) on the inflammatory (serum IL-8; chemotaxis and O_2_^−^ production by neutrophils; and IL-1β, TNF-α, IL-6, IL-10, and IL-18 release by monocytes) and stress (cortisol; NA; and eHsp72) responses in women diagnosed with FM compared with an aged-matched control group of healthy women (HW). IL-8, NA, and eHsp72 were determined by ELISA. Cytokines released by monocytes were determined by Bio-Plex® system (LUMINEX). Cortisol was determined by electrochemoluminiscence, chemotaxis was evaluated in Boyden chambers and O_2_^−^ production by NBT reduction. In the FM patients, the exercise induced a decrease in the systemic concentration of IL-8, cortisol, NA, and eHsp72; as well as in the neutrophil’s chemotaxis and O_2_^−^ production and in the inflammatory cytokine release by monocytes. This was contrary to the completely expected exercise-induced increase in all those biomarkers in HW. In conclusion, single sessions of moderate cycling can improve the inflammatory status in FM patients, reaching values close to the situation of aged-matched HW at their basal status. The neuroendocrine mechanism seems to be an exercise-induced decrease in the stress response of these patients.

## Introduction

Fibromyalgia is a form of non-articular rheumatism defined by the presence of chronic widespread pain and allodynia to pressure in more than 11 of 18 specified sites or tender points [1]. Compared with healthy women, FM patients have fatigue and stiffness and display reduced physical performance capacity, and “modified exercises” (both land-based and aquatic ones) are the main non-pharmacological interventions used for these patients, improving pain and quality of life [2]. However, the mechanisms involved in the “modified exercise”-induced benefits are still poorly understood. Although FM as an inflammatory state is not universally accepted, the current hypotheses of the aetiology of FM syndrome include a systemic and local chronic inflammatory state accompanied by an altered stress response [3]. The main objective when treating patients with inflammatory diseases is to diminish inflammation and manage secondary consequences in order to improve their quality of life [4]. Acceptance of the hypothesis of the anti-inflammatory effects of exercise [5], [6] makes it especially interesting as a therapeutic aid in treating inflammatory pathologies. Previous studies from our laboratory have found that, compared with healthy women, FM patients have higher circulating levels of IL-8, cortisol, and noradrenaline (NA), together with an increased release of pro-inflammatory cytokines by monocytes; and that these inflammatory and stress biomarkers improve after habitual aquatic exercise [7]–[9]. Nevertheless, the effects of habitual and acute exercise may be different in patients with chronic diseases and in healthy controls. It is well known that acute exercise activates the innate and inflammatory response in healthy people in terms of phagocytic function [10], and such inflammatory markers as the release of inflammatory cytokines [11]–[15]. In addition, many aspects of the exercise-induced activation of the inflammatory response are mediated by stress hormones and proteins, especially by glucocorticoids [16], catecholamines [17], and eHsp72 [18], [19], with NA and eHsp72 being of particular importance during acute sessions of moderate exercise [20], [21]. Depending on the type of exercise and disease, exacerbation and attenuation have both been observed in chronic inflammatory disease [4]. Although contraindications for exercise performance in FM patients have not been described, it is necessary to define the duration and intensity of exercise programs to obtain anti-inflammatory responses that lead to an optimized inflammatory cytokines-HPA axis-sympathetic response feedback, avoiding pro-inflammatory responses that could exacerbate the pathology [7]. This is particularly important in female, because even in their basal state healthy women have higher circulating levels of IL-8, NA, and eHsp72, as well as a greater activity of neutrophils than men [22], and these inflammatory/stress biomarkers increase after single bouts of both moderate and intense exercise [15]. Bearing this in mind, the question arises as to whether exercise, performed in single sessions, might exacerbate rather than improve the inflammatory and stress status in FM women.

The objective of the present study was to compare the effect of a single bout of moderate cycling on the inflammatory (serum IL-8; chemotaxis and O_2_^−^ production by neutrophils; and IL-1β, TNF-α, IL-6, IL-10, and IL-18 production by monocytes) and stress (cortisol, NA, and eHsp72) responses in women diagnosed with FM compared with healthy women. To the best of our knowledge, this is the first study on the effect of an acute session of exercise on the inflammatory and stress responses in FM patients.

## Methods

### Study Design and FM Patients

#### FM volunteers

8 women diagnosed with FM by a rheumatologist (at least two years before starting the investigation and according to the ACR criteria for the syndrome [1]) were enrolled in the study. All the FM patients belonged to the Fibromyalgia Associations of Badajoz (Spain). Some anthropometric, demographic, and clinical data are shown in table 1. They were requested to fill out a questionnaire about their lifestyle (diet, habits, etc.), medication, and other previous or current concomitant illnesses, and to complete the Spanish version of the Physical Activity Readiness Questionnaire (Rpar-Q) (which identifies individuals who need a medical check before exercise); as well as the “Fibromyalgia Impact Questionnaire” (FIQ), which is a specific health questionnaire that evaluates current health status in patients with FM, being 0 the optimal score. Pain was also evaluated through the SF-36 questionnaire, being 100 the optimal score. All FM volunteers were passed fit for the exercise. Total FIQ score (62±5) and pain score (24±5) were in the range of FM patients according previous data from our laboratory. All procedures were performed with the subject’s written consent. The exclusion criteria were: neoplasic illness (diagnosed from the medical history), infection, internal medical conditions (cardiopulmonary, vascular, or other), as well as the use of oral or local corticosteroids or anticytokine therapy that could influence the level of cytokines. According to the classification criteria of Müller and co-workers [23], these volunteers with FM belonged to the primary group (with no definitive organic factor triggering the syndrome), specifically to Group I (fibromyalgia with sensitivity to pain but no diagnosis of depression or other relevant psychiatric disorder). Control group: 8 age-matched healthy women (who had no pain disorders or infectious illness at the time of blood sampling) served as controls. The same requirements were applied to the control group as to the FM group.

**Table 1:** Anthropometric, demographic and clinical data.

	HW	FM volunteers
Age (years)	47±4	49±7
Weight (kg)	67±6	69±7
Body mass index (kg·m^−2^)	25±1	27±2
Hip-to-waist ratio	0.78±0.03	0.80±0.02
Body fat (%)	38±3	39±2
Time from the diagnostic (years)	–	>2*
Employment status		
Blue collar	2	3
White collar	4	2
Unemployed	2	3

HW: Healthy Women; FM: Fibromyalgia women. Data are shown as mean ± standard error.

* 100% volunteers.

All volunteers (patients and controls) were physically inactive, having undertaken no exercise program during the previous 24 months, non-smokers, and not heavy consumers of alcohol. They performed a single bout of moderate exercise at 9 a.m. local time with the participants fasting and at rest for at least 1 h before. The exercise was performed on an ergometer (Ergometrix mod. Ergo 800 S) for 45 min at each volunteer’s 55% VO_2_ max, according to Astrand and Ryhming nomogram [24] for each volunteer, previously validate in our laboratory by standard methods – ergospirometry and a heart rate monitor (Polar 5720) with an interface (Polar Advantage interface). Heart rate was monitored throughout the bout of exercise for each volunteer (Polar). The volunteers were allowed to drink water *ad libitum* during exercise.

Peripheral blood samples were drawn by antecubital vein puncture. Sampling was carried out before (basal state) and immediately after exercise in each volunteer, with all determinations performed individually. The study was approved by the Ethical Committee of the University of Extremadura (Spain) according to the guidelines of the European Community Council Directives and the Declaration of Helsinki.

### Serum and Plasma

After extraction, blood samples for serum isolation were maintained for 15–20 min at room temperature. For plasma isolation, the blood was anticoagulated with citrate during the extraction. For NA determination, 40 µl of a solution containing 900 mg of EGTA and 700 mg of glutathione in 100 ml of 0.1 M NaOH was added to 2 ml of each blood sample before plasma separation. All the samples were centrifuged at 700 *g* for 10 min, and finally the serum and plasma samples were aliquoted and stored at −80°C until assay.

### Inflammatory Cell Isolation

Blood samples were centrifuged in a density gradient (Histopaque, Sigma) obtaining a first halo containing monocytes and lymphocytes and a second one containing neutrophils. These two suspensions were washed in PBS. Isolated neutrophils were adjusted to 10^6^ cells/ml in Hank’s medium in order to evaluate their chemotaxis and O_2_^−^ production. Monocytes were purified from the *pool* of mononuclear cells using the Monocyte Isolation Kit II (Miltenyi Biotec GmbH). The monocytes were adjusted to 10^6^ cells/ml of medium (Iscove Medium [GIBCO] supplemented with 10% fœtal bovine serum, 1% penicillin/streptomycin, and 1% L-glutamine) in order to evaluate their inflammatory cytokine releasing capacity. Using this monocyte isolation technique, human monocytes were isolated by depletion of non-monocyte cells (negative selection). Non-monocytes (i.e., T cells, NK cells, B cells, dendritic cells, and basophils) were indirectly magnetically labeled with a cocktail of biotin-conjugated monoclonal antibodies (against CD3, CD7, CD16, CD19, CD56, CD123, and glycophorin A) as primary labeling reagent, and anti-biotin monoclonal antibodies conjugated to microbeads as secondary labeling reagent. The magnetically labeled non-monocyte cells were depleted by retaining them on a MACS® Column (Miltenyi Biotec GmbH, Germany) in the magnetic field of a Midi-MACS Separator (Miltenyi Biotec GmbH, Germany), while the unlabeled monocytes passed through the column. In this way, isolation of highly pure unlabeled monocytes was achieved by depletion of the magnetically labeled cells. Although a negative selection for monocytes isolation may constitute a limitation of the method, with this technique one obtains a suspension highly enriched in monocytes (higher than 90%, as determined by flow cytometry). Finally, cell viability (more than 98%) was checked by the trypan blue exclusion test.

### Determination of Systemic Inflammatory and Neuroendocrine Biomarkers

The serum concentration of IL-8 (DIACLONE) and the plasma concentrations of NA (LDN) and Hsp72 (Stressgen) were determined using commercial ELISA kits according to the procedures provided by manufacturers. The plasma concentration of cortisol was determined by electrochemoluminescence (Roche Elicys).

### Determination of Inflammatory Cytokines Released by Monocytes

Monocytes were cultured for 24 h (at 37°C, 5% CO_2_, and 100% RH) in flat-bottom 48-well cell culture plates (Falcon, Becton Dickinson Labware) in presence of bacterial lipopolysaccharide (LPS; final concentration: 50 µg/ml) [8]. Cell viability was checked by the Trypan blue exclusion test, finding at least 98% viable cells. Supernatants were aliquoted in Eppendorf tubes and stored at −80°C until assay. The release by monocytes of IL-1β, TNF-α, IL-6, IL-10, and IL-18 was evaluated using the Bio-Plex® system (LUMINEX, BioRad). This system uses fluorescently dyed beads, a flow cytometer and associated optics, and a high-speed digital signal processor to detect of up to 100 different types of molecules in a single well of a 96-well microplate. Spontaneous release of inflammatory cytokines by monocytes (in absence of LPS) was also evaluated.

### Chemotaxis

Neutrophil chemotaxis was evaluated in a Boyden chamber with a modification [25] of the original technique [26] using an isopore polyvinylpyrrolidone-free polycarbonate filter with a pore diameter of 3 µm (MILLIPORE) placed between the two compartments of the chamber. N-formyl-methionyl-leucyl-phenylalanine peptide (fMLP; 10^−8^ M, SIGMA) diluted in PBS was used as a chemoattractant in the lower compartment of the Boyden chamber. This technique is appropriate for quantitative evaluation of chemotaxis in isolated phagocytes [26]. Aliquots of 300 µl of neutrophil suspension (10^6^ cells/ml of Hank’s medium) were put into the upper compartment of the Boyden chamber and allowed to migrate through the filter for 90 min at 37°C in a 5% CO_2_ atmosphere. After incubation, the filters were removed from the chambers, fixed, and stained. The Chemotaxis Index was determined as the number of neutrophils counted at random (under phase contrast microscopy ×100) in sixteen fields of the lower face of the filter.

### Oxygen-dependent Microbicidal Capacity

The neutrophils’ oxygen-dependent microbicidal capacity (production of O_2_^−^) was evaluated by the nitroblue tetrazolium (NBT) reduction test. An aliquot of 250 µl of neutrophil suspension (10^6^ cells/ml) was incubated for 30 min with an equal volume of NBT (SIGMA, 1 mg/ml in PBS solution) in the presence of 20 µl of latex (1.09 mm diameter diluted at 1% in PBS). Aliquots of neutrophils suspension incubated in the absence of latex were used as non-stimulated samples and a solution with the same reactants and medium without neutrophils was used as blank. After 30 min of incubation the reaction was stopped with 2.5 ml hydrochloric acid (0.5 N). Samples were centrifuged for 30 min at 600 *g* and 4°C, the supernatant was discarded and the reduced NBT (formazan) extracted from the cell pellet with 1 ml of Dioxan. The tubes were then centrifuged for 30 min at 600 *g* and the absorbance of the supernatant was determined in a spectrometer at 525 nm. The results are the percentage of stimulation of the stimulated samples taking the non-stimulated as reference (100%).

### Statistical Analysis

Values are expressed as mean ± SEM. The normality of the variables was checked by Kolmogorov Smirnov normality test. Student’s paired *t*-test or unpaired t-test for normally distributed samples and Wilcoxon or Mann-Whitney test for non-normally distributed samples were used for comparing the effect of exercise in HW or FM groups separately. ANOVA was used to evaluate differences between HW and FM in the basal status and HW *vs* FM+E, in order to confirm that acute exercise do not immunocompromise FM patients; all of them according to our scientific hypothesis. The significance level was set at p<0.05.

## Results

**Figure 1:**
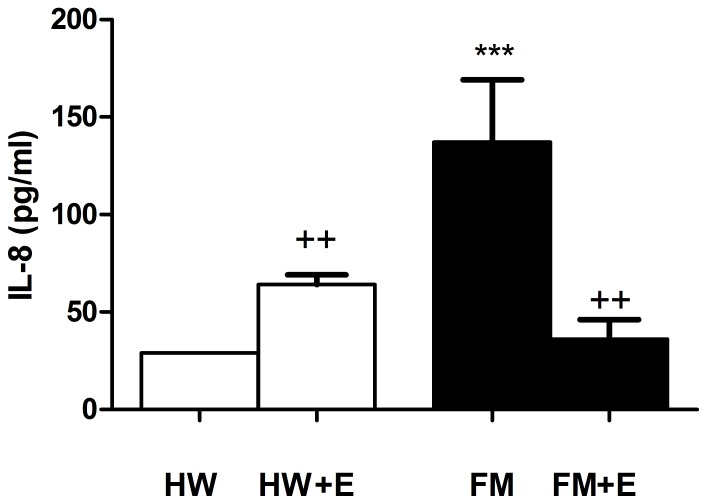
Effect of an acute session of moderate exercise (+E) on the serum concentration of IL-8 in healthy women (HW; n = 8) and in FM patients (FM; n = 8). The results are presented as the mean value ± SEM of a set of independent experiments performed in duplicate (one experiment per volunteer). ***p<0.001 with respect to HW;^++^p<0.01 with respect to each respective pre-exercise values.

**Figure 2:**
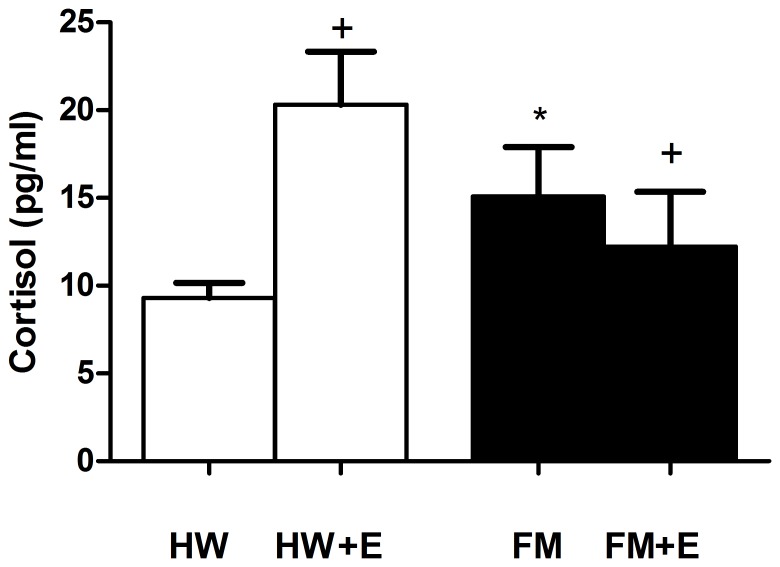
Effect of an acute session of moderate exercise (+E) on the plasma concentration of cortisol in healthy women (HW; n = 8) and in FM patients (FM; n = 8). The results are presented as the mean value ± SEM of a set of independent experiments performed in duplicate (one experiment per volunteer). *p<0.05 with respect to HW;^+^p<0.05 with respect to each respective pre-exercise values.

**Figure 3:**
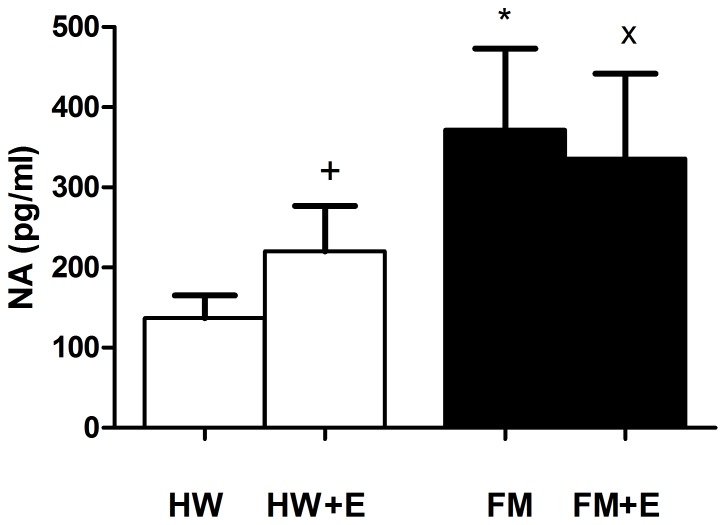
Effect of an acute session of moderate exercise (+E) on the plasma concentration of NA in healthy women (HW; n = 8) and in FM patients (FM; n = 8). The results are presented as the mean value ± SEM of a set of independent experiments performed in duplicate (one experiment per volunteer). *p<0.05 and ^x^p<0.05 with respect to HW (ANOVA);^+^p<0.05 with respect to each respective pre-exercise values.

**Figure 4:**
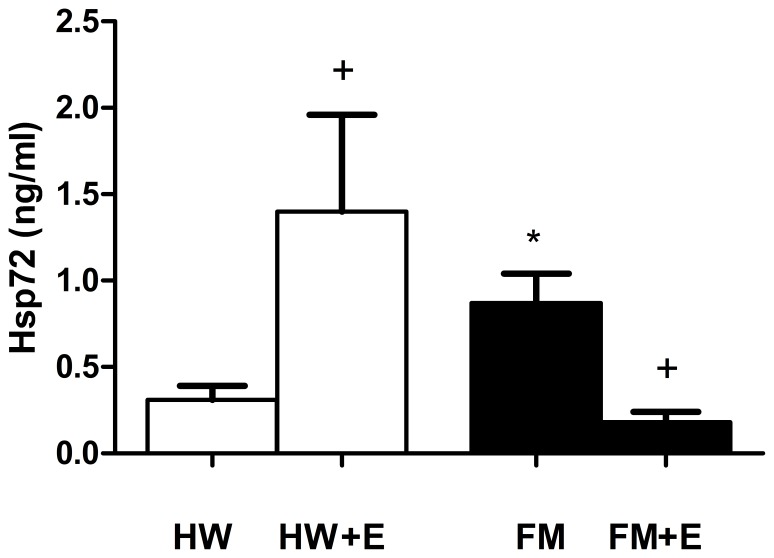
Effect of an acute session of moderate exercise (+E) on the plasma concentration of eHsp72 in healthy women (HW; n = 8) and in FM patients (FM; n = 8). The results are presented as the mean value ± SEM of a set of independent experiments performed in duplicate (one experiment per volunteer). *p<0.05 with respect to HW;^+^p<0.05 with respect to each respective pre-exercise values.

Figure 1, 2, 3, and 4 show the results corresponding to the inflammatory (IL-8) and stress (cortisol, NA, and eHsp72) biomarkers determined in serum and in plasma respectively. As expected, and consistent with previous results in a larger group of FM patients [8], the basal concentrations of all these inflammatory and stress biomarkers were significantly higher in the FM patients than in the healthy women. The effect of a single bout of moderate cycling was surprising: whereas exercise increased IL-8 (Fig. 1), cortisol (Fig. 2), NA (Fig. 3), and eHsp72 (Fig. 4) in HW, it decreased IL-8 (p<0.01), cortisol (p<0.05), NA (without the differences being significant), and eHsp72 (p<0.05) in the FM group. It was noteworthy that, after exercise, the systemic biomarkers were statistically similar to those determined in HW in the basal state (except NA).

**Figure 5:**
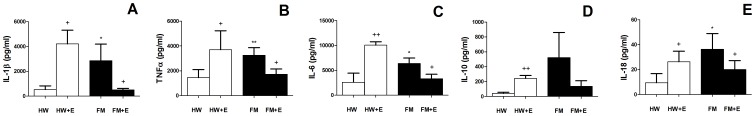
Effect of an acute session of moderate exercise (+E) on the release of cytokines by monocytes. IL-1β (A), TNF-α (B), IL-6 (C), IL-10 (D) and IL-18 (D) released by monocytes activated by LPS from healthy women (HW; n = 8) and FM patients (FM; n = 8). The results are presented as the mean value ± SEM of a set of independent experiments performed in duplicate (one experiment per volunteer). *p<0.05, **p<0.01 with respect to HW;^+^p<0.05,^++^p<0.01 with respect to each respective pre-exercise values.

Figures 5 (monocytes) and 6 (neutrophils) represent the effect of exercise on the functional capacity of inflammatory cells. In general, all the basal values determined in the group of FM patients were higher than those determined in HW. Figure 5 shows the results for the release of inflammatory cytokines by monocytes induced by LPS. The moderate exercise induced a significant increase in the release of all the cytokines (IL-1β, TNF-α, IL-6, IL-10, and IL-18) by the monocytes from HW (p<0.05 for IL-1β, TNF-α, and IL-18; p<0.01 for IL-6 and IL-10). On the contrary, the exercise induced a decrease (p<0.05) in the release of inflammatory cytokines by monocytes from FM patients (although without significant differences for IL-10).

A similar behavior was found for the spontaneous release (in absence of LPS), except for TNF-α in which no significant changes were determined in FM patients after the acute exercise.

**Figure 6:**
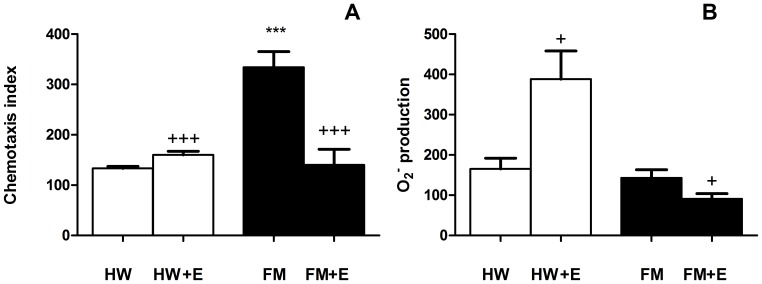
Effect of an acute session of moderate exercise (+E) on chemotaxis (A) and O_2_^−^ production (B) by neutrophils from healthy women (HW; n = 8) and FM patients (FM; n = 8). The results are presented as the mean value ± SEM of a set of independent experiments performed in duplicate (one experiment per volunteer). ***p<0.01 with respect to HW;^+^p<0.05,^+++^p<0.001 with respect to each respective pre-exercise values.

Chemotaxis (Fig. 6a) and intracellular O_2_^−^ production (Fig. 6b) were affected in the same way following exercise: an increase (p<0.001 and p<0.05, respectively) in HW and a decrease (p<0.001 and p<0.05, respectively) in FM patients.

## Discussion

Based on the hypothesis of the anti-inflammatory effects of exercise, “modified habitual exercise” is considered to be an especially good therapeutic aid in the treatment of inflammatory pathologies [5], [27], including FM [7]. However, it is not know the effects of single bouts of exercise on women with this syndrome. This takes on special relevance since this kind of exercise stimulates the inflammatory response in healthy women [15]. While a controlled stimulation of the inflammatory response induced by acute exercise can be beneficial for providing protection against infection [16], it is plausible to speculate that an over-activation of the inflammatory response could be detrimental for FM patients and exacerbate the symptoms. On the other hand, it is also possible to hypothesize, at least conceptually, that the anti-inflammatory effects of exercise are mainly or even only positive for people with an unhealthily high inflammatory status, such as individuals suffering diseases associated with chronic inflammation, such as FM. As we have previously found [7]–[9], the present results confirmed that, compared with HW, primary FM patients showed high circulating levels of IL-8, NA, cortisol, and eHsp72, together with a higher release of pro-inflammatory cytokines by monocytes and an activated status of neutrophils. Paradoxically, whereas in HW the acute moderate exercise increased the circulating levels of IL-8, cortisol, NA, and eHsp72, the neutrophils’ chemotaxis and O_2_^−^ production, and the release of inflammatory-involved cytokines (IL-1β, TNF-α, IL-6, IL-10, IL-18) by monocytes, in the FM patients the same exercise reduced all these inflammatory and stress biomarkers.

IL-8 is a pro-inflammatory cytokine mainly produced by monocytes and endothelial cells [28] which is clearly elevated in FM patients [7], [9], [29], [30]. It is involved in the symptoms of this syndrome (such as pain) and is regarded as the main systemic inflammatory marker for this pathology. Consistent with previous results [15], the acute moderate exercise increased the serum IL-8 concentration in HW. Surprisingly, however, the exercise reduced IL-8 concentration in the FM patients. These results were our first evidence that an acute session of moderate cycling could be “pro-inflammatory” in HW but “anti-inflammatory” in FM patients. This was confirmed with the results obtained for the neutrophils, since the exercise increased their chemotaxis and O_2_^−^ production (good indexes for the inflammatory and oxidative status of neutrophils) in HW, but reduced these capacities in the FM patients. This is coherent with the concept that IL-8 is a cytokine with a strong capacity to activate neutrophilś chemotaxis [28]. This opposite behaviour of neutrophilś chemotaxis in response to a session of moderate exercise between HW and the FM patients seems to be also regulated by an opposite stress response mediated by cortisol, NA, and eHsp72 as potential neuroendocrine mechanisms. Thus, all three of these stress biomarkers also presented increased circulating concentrations in the HW after the exercise, but decreased after exercise in the FM patients. Post-exercise concentrations of NA [17], eHsp72 [19], and glucocorticoids [25] mediate the exercise-induced stimulation of the chemotaxis. This suggests a role of these “stress mediators” in the opposite effects induced by the acute session of moderate exercise on neutrophils between the two experimental groups (HW *vs* FM). Indeed, it could be speculated that the apparent exercise-induced changes (although without significant differences) in the concentration of NA might contribute, at least in part, to the variations in the circulating levels of Hsp72 since, in the context of exercise-induced stress, NA increases the expression and release of Hsp72 by human neutrophils [31].

Furthermore, eHsp72 induces the release of inflammatory cytokines (such as IL-1β, TNF-α, and IL-6) by monocytes [32]. We confirmed the same effect (also for IL-18) in our HW and FM patients (data not shown). Thus, *in vivo*, while the increased circulating levels of eHsp72 after the exercise might contribute to an increased release of the inflammatory cytokines by monocytes after the exercise in HW, the exercise-induced decrease in the circulating levels of eHsp72 in women with FM might be contributing to the effect of exercise on the release of cytokines by monocytes in these patients. This potential role of eHsp72 *in vivo* might also explain, at least in part, the similar behaviour in the spontaneous release (in absence of LPS) of cytokines after exercise. During a local inflammatory response, IL-1β, TNF-α, and IL-6 are the main cytokines released by monocytes, together with the anti-inflammatory cytokine IL-10, which is released later. Compared with HW, the release of those cytokines by monocytes from FM patients is higher, contributing to their inflammatory status [8], [9]. IL-18 is also a pro-inflammatory cytokine that contributes to the organism’s systemic and local defences, and thus has been proposed as being one of the main pro-inflammatory cytokines involved in inflammatory diseases [33], [34]. The results related to the release of inflammatory cytokines by monocytes also confirmed that, although a single session of moderate exercise can be pro-inflammatory for HW, it is anti-inflammatory for FM patients. Even, the decrease in IL-10 after exercise was not so marked as determined in the pro-inflammatory cytokines. It is also important to emphasize that this anti-inflammatory effect does not seem to immune-compromise the FM patients, because their post-exercise values are close to the basal levels determined in HW.

Contrary to one of the possibilities that we initially hypothesized, the present investigation failed to find an increase in the inflammatory and stress responses induced by a single session of exercise in the FM patients, an increase that would potentially exacerbate their symptoms. Considering the results as a whole, one may conclude that a single session of moderate cycling induced an anti-inflammatory effect in FM patients.

Another reflection that comes from the present investigation is that responses to exercise could differ according to each individual’s particular set-point, and that, even though two individuals might show opposite responses, these responses may each be working in a positive regulatory direction. On the one hand, the activation of the innate and/or inflammatory responses (considered to be an “alert state”) is indispensable for avoiding and defending against pathogenic attacks, and could be interpreted in HW as a positive physiological adaptation in a situation of vulnerability for the organism [35]. On the other hand, the anti-inflammatory effects induced by moderate exercise in FM patients can also be considered a positive effect of exercise, with the focus now being on homeostatic adjustment, with inflammation-stress feedback mechanisms being involved. Nevertheless, we cannot be sure if this anti-inflammatory and stress responses induced by acute sessions of moderate exercise is exclusive for FM patients, or exercise can affect in the same way to other inflammatory and stress pathologies. In a very interesting review evaluating the effects of acute exercise in most of the chronic inflammatory diseases compared to healthy people, Ploeger et al. [4] find in general, similar inflammatory response (generally increased) after exercise in both healthy people and patients with chronic inflammatory diseases (asthma, arthritis, chronic heart failure, chronic kidney disease, cystic fibrosis, diabetes mellitus, inflammatory bowel disease, McArdlés disease, myositis, and multiple sclerosis). The sample size of patients might be considered as a potential limitation of the present study, since different conditions of FM patients (such as obesity and others) could potentially produce variations in terms of inflammatory parameters among different studies. Nevertheless, the results were very consistent in all the patients, a fact that in our opinion can reinforce the conclusions of the present work. In addition, to the best of our knowledge, this is the first time that a “big set” of stress and inflammatory biomarkers are evaluated, with respect to exercise, in each FM patient individually.

## Conclusions

Single sessions of moderate cycling improve the inflammatory and stress status of FM patients. Thus, adaptations induced by “modified moderate cycling” could be a good non-pharmacological therapeutic help for primary FM patients.

In addition, the results of the present investigation clearly suggest that the inflammatory response after acute exercise can differ according to each individuaĺs set point mediated by exercise-induced stress mediators.
